# Auditing the Immunization Data Quality from Routine Reports in Shangyu District, East China

**DOI:** 10.3390/ijerph13111158

**Published:** 2016-11-18

**Authors:** Yu Hu, Xinpei Zhang, Qian Li, Yaping Chen

**Affiliations:** 1 Institute of Immunization and Prevention, Zhejiang Provincial Center for Disease Control and Prevention, Hangzhou 310021, China; husix@163.com (Y.H.); qianli@cdc.zj.cn (Q.L.); 2 Shangyu District Center for Disease Control and Prevention, Shangyu 312300, China; zxp8790@163.com

**Keywords:** audit, data quality, immunization, coverage, monitoring

## Abstract

**Objective:** To evaluate the immunization data quality in Shangyu District, East China. **Methods:** An audit for immunization data for the year 2014 was conducted in 20 vaccination clinics of Shangyu District. The consistency of immunization data was estimated by verification factors (VFs), which was the proportion of vaccine doses reported as being administered that could be verified by written documentation at vaccination clinics. The quality of monitoring systems was evaluated using the quality index (QI). **Results:** The VFs of 20 vaccine doses ranged from 0.94 to 1.04 at the district level. The VFs for the 20 vaccination clinics ranged from 0.57 to 1.07. The VFs for Shangyu District was 0.98. The mean of total QI score of the 20 vaccination clinics was 80.32%. A significant correlation between the VFs of the 3rd dose of the diphtheria–tetanus–pertussis combined vaccine (DTP) and QI scores was observed at the vaccination clinic level. **Conclusions:** Deficiencies in data consistency and immunization reporting practice in Shangyu District were observed. Targeted measures are suggested to improve the quality of the immunization reporting system in vaccination clinics with poor data consistency.

## 1. Introduction

Immunization is one of the most important measures of controlling vaccine-preventable diseases and has been considered the most cost-effective public health intervention. Immunization is provided through expanded programs on immunization (EPIs) as a part of primary health care in many countries, including China [1]. 

The monitoring of vaccination coverage is one of the most important components of an EPI. Coverage estimates are usually based on two different resources of empirical data: administrative data and coverage survey. For estimates based on administrative data, coverage is derived by dividing the total number of vaccination given by the number of target population. Most countries track the performance of EPIs with a hierarchical administrative reporting system, as it is more convenient than estimates based on surveys [2]. Administrative monitoring systems can provide a strong basis from which to guide planning, review progress, and address gaps in areas with low-coverage or high drop-out rates; if accurate, timely reporting occurs at each level. However, administrative data collected through routine immunization reporting system have been criticized for inaccuracy [3]. Several studies have reported inconsistencies in data reporting. For example, a study done in Nepal [2] found that data obtained from the vaccination clinic were lower than the data reported to the district level, showing a tendency of over-reporting to higher levels. Another study [4] showed errors in reporting due to the lack of supervision and feedback, as well as inadequate incentives to vaccination staff. 

China started an EPI in 1978 with 4 vaccines, and as of now this program continues with 11 vaccines. Under the national law on prevention and cure of contagious diseases, all EPI vaccines are free and mandatory. According to the Chinese EPI [1], “A fully vaccinated child under 7 years of age” takes one dose of the Calmette–Guerin vaccine (BCG), two doses of the oral polio live attenuated vaccine (OPV), four doses of the diphtheria–tetanus–pertussis combined vaccine (DTP), one dose of the diphtheria–tetanus combined vaccine (DT), one dose of the measles–rubella combined live attenuated vaccine (MR), one dose of the measles–mumps–rubella combined live attenuated vaccine (MMR), three doses of the hepatitis B vaccine (HepB), one dose the hepatitis A live attenuated vaccine (HepA), two doses of the meningococcal polysaccharide vaccine type a (M-a), two doses of the meningococcal polysaccharide vaccine types a and c (M-ac), and two doses of Japanese encephalitis live attenuated vaccine (JEV). In China, staff at vaccination clinics compile vaccination data from daily immunization logs, tally sheets, or an immunization information system and report these data to the district level center for disease control and prevention (CDC) monthly. The district level CDC aggregates the coverage data from all vaccination clinics and reports them to the provincial level monthly. Ideally, the staff of CDCs at different levels uses these reports to assess the performance of the EPI. To verify the quality of administrative monitoring systems, the World Health Organization (WHO) developed evaluation protocols known as the immunization data quality audit (DQA) [5,6]. 

To improve the quality of immunization data, a two-week assessment study based on DQA protocols was implemented in 2014 in Shangyu District, East China. This report presents the results of this data quality audit and determines whether there were deficiencies in the current coverage reporting system.

## 2. Methods

### 2.1. Selection of Research Sites

Shangyu District has an estimated population of 779,892 people, with an estimated 5466 births per year based on annual population growth of 7.01%. Shangyu District was chosen for the provincial CDC, which has identified constraining predictors associated with the functionality of the EPI, such as the periodic appearance of measles epidemics that occurs in Shangyu District, with a sustained high reporting coverage of measles containing vaccine (>95%) during the last decade. The district’s health infrastructure consists of 20 vaccination clinics, all of which were selected for this study. 

### 2.2. Immunization Information System

All 20 vaccination clinics of Shangyu District participate in the Zhejiang provincial immunization information system (ZJIIS). ZJIIS is a computerized, population-based immunization registry and maintains demographic and vaccination data for children aged <7 years since 2004. The data collection, function, and management mechanism of ZJIIS can be found in [7].

### 2.3. Evaluation Process

An evaluation team involving 16 immunization staff members from a Zhejiang provincial CDC conducted this data audit in Shangyu District from 3 to 18 August 2014. The evaluation team was trained at a Chinese CDC, and the evaluation process basically complied with the recommended protocols, which called for on-site evaluations at each vaccination clinic. 

### 2.4. Evaluation Measures and Data Analysis

Two key performance measures were reviewed in this evaluation. Verification factors (VFs) constitute one of these measures [6]. VFs are vaccination clinic-based indicators of reporting consistency. For the sake of anonymity, the names of vaccination clinics were presented as VC_1_, VC_2_, VC_3_, …, VC_20_. The VF was calculated for performance for the year 2014. For each vaccination clinic, the total number of vaccine doses administrated in 2014 was re-counted from ZJIIS. The VF was defined as the value of the re-counted number divided by the number of relevant vaccination doses reported by the vaccination clinic to the Shangyu District CDC. The BCG and the first dose of HepB were not included in the analysis of VFs, as these two doses were administrated by maternity hospitals rather than vaccination clinics. 

Four different kinds of VFs were calculated, including (1) the VF for the 20 vaccine doses for each vaccination clinic (Equation (1)); (2) the VF for the 20 vaccine doses at the district level (Equation (2)); (3) the VF for the 20 vaccination clinics (Equation (3)); and (4) the VF for Shangyu District (Equation (4)). The VF at the district level was calculated as the weighted average of the VF of the vaccination clinics. The weight of each vaccination clinic was estimated by their number of total vaccinations reported in 2014. 

We use the index i = {1, …, 20} to designate the vaccination clinics and j = {1, …, 20} to designate vaccine doses, and the equations for calculating VFs were as follows:

(1) $${\text{VF}}_{i}^{j}=\frac{\text{the re}-\text{counted number of}\;j^{th}\;\text{vaccine dose administrated of the}\;i^{th}\;\text{vaccination clinic}}{\text{the reported number of specific vaccines dose of the}\;i^{th}\;\text{vaccination clinic}}$$

(2) $${\text{VF}}_{d}^{j}=\overset{20}{\underset{i=1}{\sum }}\left({\text{VF}}_{i}^{j}\times \frac{\text{the number of total vaccinations reported by the}\;i^{th}\;\text{vaccination clinic}}{\text{the number of total vaccinations reported by Shangyu District CDC}}\right)$$

(3) $${\text{VF}}_{i}=\sum_{j=1}^{20}{\text{VF}}_{i}^{j}\times \left(\frac{1}{20}\right)$$

(4) $${\text{VF}}_{d}=\sum_{i=1}^{20}{\text{VF}}_{i}\times \frac{\text{the number of total vaccinations reported at}\;i^{th}\;vaccination\;clinic}{\text{the number of total vaccinations reported in Shangyu District}}.$$

A VF of <1 indicates that lower numbers of vaccinations are recorded as administered at the vaccination clinic compared with those reflected in the file records sent to Shangyu District CDC (over-reporting). Conversely, VF of >1 indicates that higher numbers of doses are recorded as being administered at the vaccination clinic compared with those reflected in the file records sent to Shangyu District CDC (under-reporting). To characterize reporting consistency, the VF was classified into three categories in this study: (1) consistent if the VF was between 85% and 115%; (2) under-reporting if the VF was >115%; and (3) over-reporting if the VF was <85% [6]. The proportions of VF as consistent, under-reporting, and over-reporting were calculated, and the proportions of vaccination clinics with VF as consistent, under-reporting, and over-reporting were calculated for each vaccine dose.

The second key measure is the quality index (QI) [6], a quantitative indicator of the quality of the vaccination clinics. The QI is based on 22 questions or observations at the vaccination clinic level. These questions or observations are categorized into 4 components: recording practices (14 questions), storage and reporting practices (4 questions), monitoring and evaluation (2 questions), the denominators used (2 questions). One point was given for each question answered correctly or for each task observed to have been performed correctly. The QI score was defined as the number of correct answers and correctly performed tasks divided by the total number of answers and observations. For each vaccination clinic, the total QI score and the QI score, and all 4 components, were calculated separately. The mean of the total QI score was calculated as the weighted average of the QI score of the vaccination clinics. The weight of each vaccination clinic was estimated by the number of total vaccinations reported in 2014. To provide composite information on the strengths and weaknesses of the immunization reporting system, the corrective response rate for each question, and the observation in QI score assessment was also calculated. 

In order to verify the association between reporting performance and data consistency, the Spearman correlation analysis was used to explore the association between the VF of DTP_3_ and the reporting coverage of DTP_3_, and the association between the VF of DTP_3_ and the QI at the vaccination clinic level according to the experience of previous reports [6]. Accounting for the fact that VF was not a linear concept, VF > 1 was transformed by subtracting the excess (greater than 1) from 1 [1-(VF-1)] when the correlation coefficients and *p* values were calculated [6]. All analysis was performed with SPSS version 13.0 (SPSS Inc., Chicago, IL, USA) and at a significance level of 0.05. 

## 3. Results

### 3.1. Reporting Consistency

The VFs of 20 vaccine doses ranged from 0.94 to 1.04 at the district level. The VFs for the 20 vaccination clinics ranged from 0.57 to 1.07. The VF for Shangyu District was 0.98 (Table 1). 

**Figure 1:**
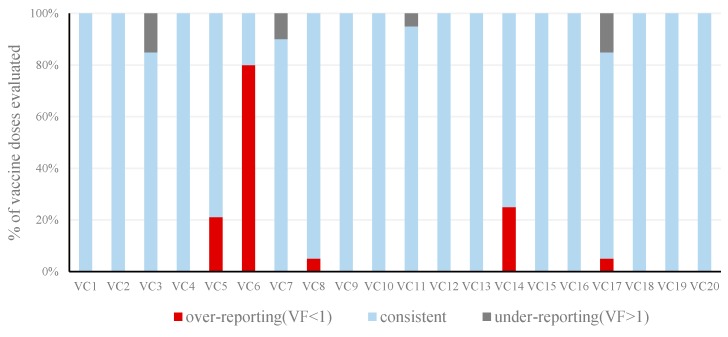
Distribution of verification factors (VFs) of the 20 vaccine doses among 20 vaccination clinics in Shangyu District, 2014.

Among the 20 vaccination clinics evaluated, 12 (60%) vaccination clinics had consistent data for each of the 20 vaccine doses; 4 (20%) vaccination clinics had under-reporting data; 3 (15%) vaccination clinics had over-reporting data; 1 (5%) vaccination clinic had both under-reporting and over-reporting data (Figure 1). 

**Figure 2:**
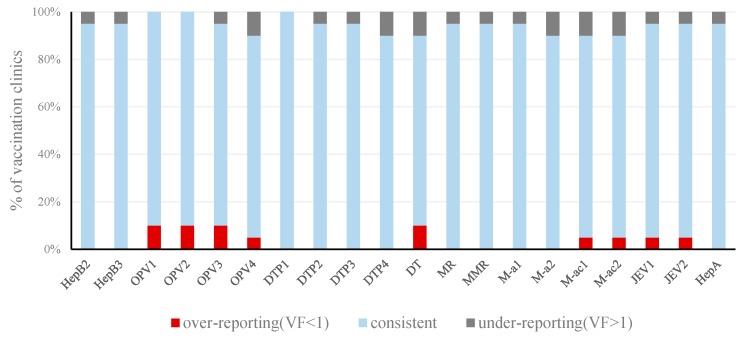
Distribution of vaccination clinics by VFs of the 20 vaccine doses in Shangyu District, 2014.

Among the 20 vaccine doses assessed, the VF ranged from 0.07 to 3.25 among 20 vaccination clinics. Only DTP_1_ had consistent data in all 20 vaccination clinics. Nine vaccine doses (four doses of OPV and DT, two doses of MPV-ac, and two doses of JEV) had under-reporting data, and 17 vaccine doses (HepB_2_, HepB_3_, OPV3, OPV4, DTP_2_, DTP_3_, DTP_4_, DT, MR, MMR, two doses of MPV-a, two doses of MPV-ac, two doses of JEV, and HepA) had over-reporting data (Figure 2).

### 3.2. Quality of Immunization Reporting System

The mean of the total QI score of the 20 vaccination clinics is 80.32%. Means of the QI score for each component varied from 65.00% to 93% (Table 2). 

Among specific questions or observations of the “recording practice” component, only 50% of vaccination clinics had an up-to-date ledger of entries, and 60% of vaccination clinics had a log of the receipt/issuing of syringes supplies. Forty percent of vaccination clinics did not have procedures for reporting adverse events. We also found substantial deficiency in giving vaccinations and appointing the next vaccination day correctly, with a correct response rate of 60% and 55%, respectively. The greatest weakness among the questions or observations of the “monitoring and evaluation” component was that none of the 20 vaccination clinics had any interaction with the local community for social mobilization or to motivate parents to vaccinate their children. The main issue of the “storage and reporting practices” component was that four vaccination clinics did not have a location to properly store historical reports and vaccination records. Within the final component “denominators,” five vaccination clinics did not have a target number of infants for receiving vaccinations against DTP, and four vaccination clinics did not know the number of new births and did not follow up these newborns (Table 3).

### 3.3. Correlation Analysis

**Figure 3:**
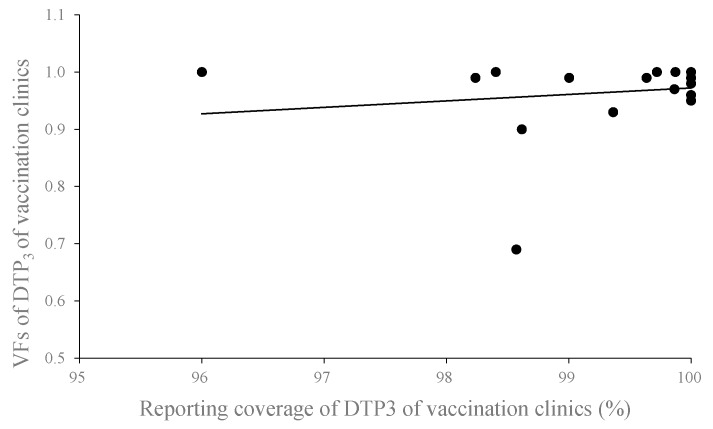
Correlation between the VF of DTP_3_ (diphtheria–tetanus–pertussis combined vaccine) and the reporting coverage of DTP_3_ at the vaccination clinic level from the data quality audit (DQA) in Shangyu District, 2014.

No significant correlation between the VF of DTP_3_ and the reporting coverage of DTP_3_ at the vaccination clinic level was observed, with a Spearman correlation coefficient of 0.399 (*p* = 0.082) (Figure 3).

**Figure 4:**
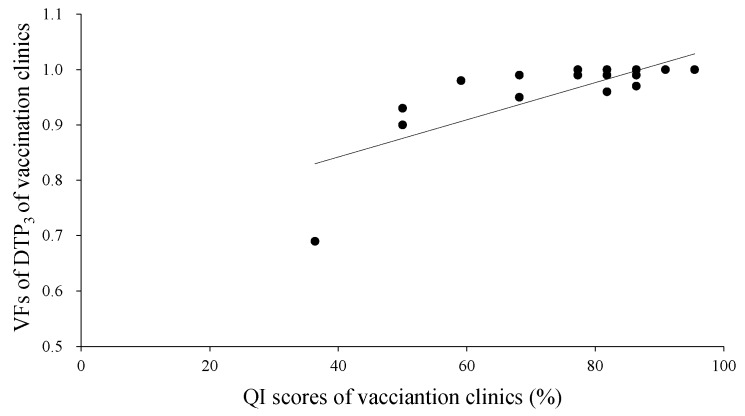
Correlation between the VF of DTP_3_ and the quality index (QI) score at the vaccination clinic level, from the DQA in Shangyu District, 2014.

Figure 4 showed a strong correlation between the VF of DTP_3_ and the QI score at the vaccination clinic level, with Spearman correlation coefficients of 0.627 (*p* = 0.003).

## 4. Discussion

An immunization data audit based on a DQA provides a quantitative index of immunization data consistency and quality. It can motivate the monitors of the immunization reporting system to make improvements on data quality. Moreover, the immunization data audit can also diagnose specific weaknesses that, if addressed, would improve the precision, efficiency, and usefulness of the immunization monitoring system. 

Several methods have been used to monitor vaccination coverage, each with their own advantages and disadvantages [8]. First, an immunization information system (such as ZJIIS) can give complete and accurate real-time data on vaccination coverage based on individual records, even though there are many challenges to their implementation, such as adequate funding and a difficulty in identifying potential duplicate records [9]. Second, administrative reporting coverage (vaccinations administered divided by the estimated target population) is often used in many developing countries, but it is considered unreliable due to an incomplete or inaccurate numerator or denominator, and delayed or duplicate reporting [10]. Third, a community-based coverage survey has been traditionally used as a method to verify reporting coverage rate and obtain a point estimate of coverage, and has also been considered as a substitute for administrative reporting coverage. However, a coverage survey can’t be conducted frequently, as it is expensive and time-consuming, so the survey results cannot reflect the real-time conditions [11]. Furthermore, a coverage survey may be subjected to selection bias and recall bias, and hardly provides information on the quality of the data or identifies weaknesses of the monitoring system [12]. Therefore, an administrative reporting system is still needed to provide critical information on an ongoing basis to determine whether coverage targets are being achieved, and we suggest that an immunization data audit be integrated with rapid assessments or supportive supervision visits routinely at the peripheral level to improve the data quality of administrative reporting coverage. Additionally, the reaching every district approach [13], recommended by the WHO, calls for strengthening the monitoring capacity and promotes the use of immunization data for planning.

This was the first time that a standard method was applied to evaluate the quality of administrative coverage reporting systems in East China. Basically, this study provided an optimal data consistency, and 60% of vaccination clinics had consistent data, which is a higher percentage than that of previous reports [6,14]. This result can be attributed to the key advantages of ZJIIS, which include (1) the ability to collect immunization information on the spot at the time of vaccination (failure to enter vaccination information into the system is therefore unlikely); (2) logic check functions to ensure the accuracy of data; and (3) long-time retention of vaccination records. 

VFs of Shangyu District ranged from 0.94 to 1.04 for different vaccine doses, which were higher than the assessment results from Nepal [2]. VFs varied widely from 0.07 for M-ac_1_ to 3.25 for DT at the vaccination clinic level, suggesting that there were errors in the coverage reporting system at the vaccination clinic level. The common reasons for under- or over-reporting may include a lack of training or feedback from a higher level, no concern on the quality of the reports, and no cross-checking mechanism. Furthermore, concerns only on timely reporting rather than accuracy at the district level, and a frequent transfer of vaccination staff, may also make the reports inaccurate. Moreover, the inaccuracy problem was more frequent due to over-reporting rather than under-reporting. We speculate two possible reasons for the over-reporting. First, all vaccination clinics need to meet the coverage goal set by the Chinese EPI, and the performance assessment of an EPI is largely based on achievements such as immunization coverage goals. As such, the coverage may be over-reported when the vaccination clinics have difficulties meeting their goals in practice. For example, the catchment area of the vaccination clinic that has a large amount of migrant people and a low compliance of vaccination may decrease the vaccination coverage. Vaccination clinics located in remote areas sometimes may make the vaccination inaccessible and induce a low coverage. Second, shortage or temporary posting of vaccination staff may have made it even more difficult to meet the coverage goals due to the poor capacity of the fresh vaccination staff. In any of these two conditions, over-reporting would be an effective strategy to satisfy higher level institutions, and this phenomenon would be more common in remote or hilly areas.

Several interventions can be applied to improve the data quality [15,16]. First, providing adequate and timely feedback through review meetings, which can help the vaccination staff focus on the data quality and monitor their own work, leading to a sense of ownership of the generated information. Second, conducting supervision visits including on-site trainings on basic concepts and monitoring indicators. Furthermore, we assume that the introduction of the automatic generation of coverage reports on the basis of ZJIIS may also improve the accuracy of reporting coverage at the vaccination clinic level.

In this study, there was no significant correlation between reporting coverage and VFs. This suggests that all vaccination clinics, regardless of coverage levels, have substantial inconsistencies in reporting data and benefit from an immunization data audit. 

In this study, we found that the quality of the reporting system was an important determinant of data consistency at the vaccination clinic level. The questions or observations that seemed to most affect the QI score were “correct vaccination date,” “correct appointment date for vaccination,” “up-to-date recording for vaccine receipt or issuing,” and “poor interaction with communities.” These results reflected that vaccination workers’ knowledge or capacity with respect to immunization might be inadequate, affecting their routine tasks of immunization, including the coverage reporting. Thus, we suggest that the Shangyu District CDC provide a targeted refreshing training for all vaccination staff from vaccination clinics with low VFs or QI scores. The training content should focus on valid immunization, correct appointment, vaccine stock registry, community interaction, and social mobilization. Furthermore, the relationship between a vaccination clinic and the community needs to be strengthened by establishing mechanisms of information exchange. 

The limitation of this study is the imprecision of the audit results due to the small sample size; however, increasing the sample size would increase cost and time in turn. Despite this existing limitation, we still believe that the present results constitute valuable contributions applicable to the local EPI and other settings where data inaccuracy is an obstacle to a good coverage reporting system as well as scientific policy-making.

## 5. Conclusions

This study suggested that auditing the quality of immunization reporting systems is suitable for our condition and indicated there were deficiencies in data consistency and immunization reporting practices in Shangyu District. Necessary steps, such as implementing review meetings, refreshing training, supervision visits, and strengthening relationships between vaccination clinics and the community, are suggested to further increase the accuracy of immunization data and the quality of the immunization reporting system.

## Figures and Tables

**Table 1:** District and vaccination clinic VFs from audit of immunization data quality in Shangyu District, 2014.

Unit *	Weight	HepB_2_	HepB_3_	OPV_1_	OPV_2_	OPV_3_	OPV_4_	DTP_1_	DTP_2_	DTP_3_	DTP_4_	DT	MR	MMR	M-a_1_	M-a_2_	M-ac_1_	M-ac_2_	JEV_1_	JEV_2_	HepA	VF of Unit
VC_1_	0.23	0.99	0.95	0.99	1.03	0.99	0.98	1.03	1.03	1.03	1.00	0.94	0.94	0.99	0.99	0.99	0.94	0.99	0.94	0.98	1.00	0.99
VC_2_	0.12	0.97	0.97	1.01	1.01	1.01	0.99	1.01	1.00	1.00	1.00	0.94	0.92	0.94	1.00	0.96	1.00	0.91	0.97	0.96	0.87	0.97
VC_3_	0.04	1.02	1.00	1.44	1.35	1.31	1.00	1.12	1.12	1.10	1.00	0.96	1.01	0.99	1.01	0.97	1.02	0.99	0.95	0.94	1.01	1.07
VC_4_	0.04	0.99	0.93	0.98	1.01	0.94	0.98	1.00	0.95	1.01	0.98	0.99	1.02	0.93	0.99	1.00	0.99	1.00	0.87	0.94	0.98	0.97
VC_5_	0.05	1.01	1.00	1.28	1.23	1.16	1.00	1.00	1.00	1.00	1.00	1.00	1.00	1.00	1.00	1.00	1.00	1.00	0.97	1.21	1.00	1.04
VC_6_	0.02	1.13	0.69	0.93	0.91	0.70	0.24	0.92	0.77	0.69	0.39	0.20	0.52	0.32	0.59	0.36	0.55	0.14	0.50	0.47	0.38	0.57
VC_7_	0.01	0.96	0.98	0.96	0.94	0.94	1.09	0.98	0.96	0.96	0.98	3.25	0.89	0.96	1.15	1.00	0.91	2.69	1.04	1.03	0.97	1.18
VC_8_	0.01	1.02	1.00	1.02	1.00	1.00	1.03	1.00	1.00	1.00	1.00	1.00	1.02	1.02	0.98	1.00	1.05	0.83	1.00	0.94	0.98	0.99
VC_9_	0.00	1.00	1.00	1.00	1.00	1.00	1.00	1.00	1.00	1.00	1.00	1.00	1.00	1.00	1.00	1.00	1.00	1.00	1.00	1.00	1.00	1.00
VC_10_	0.00	0.89	1.05	1.10	0.96	0.93	1.00	1.04	0.96	1.04	0.97	1.05	1.04	0.93	1.00	0.97	0.96	0.98	0.97	1.00	0.98	0.99
VC_11_	0.06	1.02	0.99	1.07	1.05	1.03	1.00	1.01	1.01	1.00	1.01	0.95	1.09	1.00	1.00	1.01	1.00	0.96	1.28	1.29	1.01	1.04
VC_12_	0.01	0.99	1.01	1.01	1.00	1.00	1.00	1.00	1.00	1.00	1.01	1.00	1.00	0.99	1.01	1.01	1.01	0.94	1.00	0.99	1.03	1.00
VC_13_	0.03	1.10	0.99	1.01	1.01	1.01	0.99	1.00	1.01	1.00	1.01	1.03	1.00	1.01	1.00	1.01	1.00	1.07	1.01	1.01	1.01	1.01
VC_14_	0.02	0.79	0.85	0.93	0.91	0.92	0.54	0.92	0.85	0.93	0.91	0.50	0.94	0.94	0.91	0.82	0.07	0.97	0.85	0.85	1.05	0.82
VC_15_	0.03	0.99	1.00	1.00	0.98	1.00	0.98	0.98	1.01	0.98	1.00	1.01	1.06	1.00	0.99	1.00	1.01	0.97	0.99	0.99	0.99	1.00
VC_16_	0.02	0.95	1.00	1.00	1.01	1.01	0.96	1.00	1.01	1.01	1.00	0.98	1.01	0.99	1.01	0.99	1.01	1.00	1.01	1.00	0.99	1.00
VC_17_	0.05	1.02	1.03	1.01	1.01	1.02	1.29	1.00	1.00	1.01	0.78	1.20	1.02	1.06	1.00	1.01	1.19	1.10	1.04	1.19	1.10	1.05
VC_18_	0.03	0.96	0.94	0.97	0.94	0.96	0.96	0.95	0.98	0.95	0.98	0.99	0.96	0.99	0.94	1.00	0.97	0.98	0.98	0.99	0.98	0.97
VC_19_	0.06	1.00	1.00	1.00	1.00	1.01	1.00	1.01	1.01	0.99	1.00	1.00	1.01	0.99	1.00	1.00	1.02	1.00	1.04	1.00	1.00	1.00
VC_20_	0.16	1.00	1.00	1.08	1.04	1.01	1.00	1.00	1.00	1.00	1.00	1.00	1.01	1.00	1.10	1.05	1.00	1.00	1.00	0.99	1.00	1.01
District	-	0.99	0.95	1.04	1.03	1.02	0.96	1.00	1.00	0.99	0.97	0.94	0.96	0.96	0.99	0.98	0.96	0.95	0.97	1.00	0.98	0.98

* VC: vaccination clinic; HepB: hepatitis B vaccine; OPV: oral polio live attenuated vaccine; DTP: diphtheria–tetanus–pertussis combined vaccine; MR: measles–rubella combined live attenuated vaccine; MMR: measles–mumps–rubella combined live attenuated vaccine; JEV: Japanese encephalitis live attenuated vaccine; HepA: hepatitis A live attenuated vaccine; VF: verification factor; District: Shangyu District.

**Table 2:** QI (quality index) scores by vaccination clinics and components from the data quality audit (DQA) in Shangyu District, 2014.

Unit *	QI Score (%)				
	Total	Recording Practices	Storage and Reporting Practices	Monitoring and Evaluation	Denominators
VC_1_	86.36	85.71	75.00	100.00	100.00
VC_2_	81.82	78.57	75.00	100.00	100.00
VC_3_	50.00	57.14	25.00	50.00	50.00
VC_4_	77.27	78.57	75.00	100.00	50.00
VC_5_	86.36	85.71	75.00	100.00	100.00
VC_6_	36.36	50.00	0.00	50.00	0.00
VC_7_	81.82	85.71	50.00	100.00	100.00
VC_8_	90.91	92.86	75.00	100.00	100.00
VC_9_	77.27	85.71	25.00	100.00	100.00
VC_10_	81.82	85.71	75.00	100.00	50.00
VC_11_	95.45	100.00	75.00	100.00	100.00
VC_12_	90.91	92.86	75.00	100.00	100.00
VC_13_	90.91	92.86	75.00	100.00	100.00
VC_14_	50.00	50.00	25.00	100.00	50.00
VC_15_	59.09	71.43	25.00	50.00	50.00
VC_16_	81.82	78.57	75.00	100.00	100.00
VC_17_	86.36	85.71	75.00	100.00	100.00
VC_18_	68.18	85.71	25.00	50.00	50.00
VC_19_	68.18	71.43	50.00	100.00	50.00
VC_20_	90.91	92.86	75.00	100.00	100.00
Mean	80.32	80.17	65.00	93.00	86.00

* VC: vaccination clinic.

**Table 3:** Questions or observations for QI evaluation at the vaccination clinic level.

Component (Number of Questions or Observations)	Number	Question or Observations	Correct Response Rate (%)
Recording Practices (14)	1	Are registers used for recording individual information about child immunization?	100.00
	2	Can a child’s vaccination history be easily and rapidly retrieved in the registry?	85.00
	3	Did every person taking the child health card exercise get a perfect score for DTP_1_ < 1?	80.00
	4	Did every person taking the child health card exercise get a perfect score for DTP_3_ < 1?	90.00
	5	Did every person taking the child health card exercise get a perfect score for measles containing vaccine < 1?	90.00
	6	Was the correct vaccination given for every vaccination observed?	60.00
	7	Was the correct date to return given for every vaccination observed?	55.00
	8	Are the individual recording forms available for the entire audit year?	100.00
	9	Are vaccination staff aware of standard operating procedure and there forms to complete if there is a report of AEFI?	60.00
	10	Does the vaccination clinic use/maintain a ledger/stock control for vaccines?	100.00
	11	Is the ledger up-to-date in entries for DTP?	50.00
	12	Is the receipt of DTP in the ledger complete for the entries audit year?	95.00
	13	Is there a log of the receipt/issuing of syringes supplies?	60.00
	14	Does the vaccination clinic record vaccine batch number and expiry date?	100.00
Monitoring and Evaluation (4)	15	Does the vaccination clinic monitor vaccine wastage?	75.00
	16	Is there interaction with the community regarding immunization?	0.00
	17	Is there a mechanism in place to track vaccine doses that are due or track defaulters?	70.00
	18	Does the vaccination clinic monitor dropout rate?	80.00
Storage and Reporting Practices (2)	19	Are all the vaccination reports available for the entire audit year?	100.00
	20	Is there one location where reports and records before 2000 year concerning immunization data are stored appropriately?	80.00
Denominators (2)	21	Does the vaccination clinic have a number of infants that they strive to vaccinate against DTP during a calendar year/reporting period/vaccination session?	75.00
	22	Is the vaccination clinic aware of new births in the catchment area and attempts to follow up to ensure all newborns are immunized?	80.00
